# Advanced Cancer Imaging Applied in the Comparative Setting

**DOI:** 10.3389/fonc.2020.00084

**Published:** 2020-02-07

**Authors:** David M. Vail, Amy K. LeBlanc, Robert Jeraj

**Affiliations:** ^1^Department of Medical Sciences, School of Veterinary Medicine, University of Wisconsin-Madison, Madison, WI, United States; ^2^Carbone Cancer Center, University of Wisconsin-Madison, Madison, WI, United States; ^3^Comparative Oncology Program, Center for Cancer Research, National Cancer Institute, Bethesda, MD, United States; ^4^Department of Medical Physics, School of Medicine and Public Health, University of Wisconsin-Madison, Madison, WI, United States

**Keywords:** canine, cancer, imaging, radiomics, biomarkers, PET/CT, theranostics, comparative

## Abstract

The potential for companion (pet) species with spontaneously arising tumors to act as surrogates for preclinical development of advanced cancer imaging technologies has become more apparent in the last decade. The utility of the companion model specifically centers around issues related to body size (including spatial target/normal anatomic characteristics), physical size and spatial distribution of metastasis, tumor heterogeneity, the presence of an intact syngeneic immune system and a syngeneic tumor microenvironment shaped by the natural evolution of the cancer. Companion species size allows the use of similar equipment, hardware setup, software, and scan protocols which provide the opportunity for standardization and harmonization of imaging operating procedures and quality assurance across imaging protocols, imaging hardware, and the imaged species. Murine models generally do not replicate the size and spatial distribution of human metastatic cancer and these factors strongly influence image resolution and dosimetry. The following review will discuss several aspects of comparative cancer imaging in more detail while providing several illustrative examples of investigational approaches performed or currently under exploration at our institutions. Topics addressed include a discussion on interested consortia; image quality assurance and harmonization; image-based biomarker development and validation; contrast agent and radionuclide tracer development; advanced imaging to assess and predict response to cytotoxic and immunomodulatory anticancer agents; imaging of the tumor microenvironment; development of novel theranostic approaches; cell trafficking assessment via non-invasive imaging; and intraoperative imaging to inform surgical oncology decision making. Taken in totality, these comparative opportunities predict that safety, diagnostic and efficacy data generated in companion species with naturally developing and progressing cancers would better recapitulate the human cancer condition than that of artificial models in small rodent systems and ultimately accelerate the integration of novel imaging technologies into clinical practice. It is our hope that the examples presented should serve to provide those involved in cancer investigations who are unfamiliar with available comparative methodologies an understanding of the potential utility of this approach.

## Introduction

Historically, cancer imaging in comparative species (e.g., pet dogs and cats) followed (lagged) behind cancer imaging technology development in humans. That is, as a new modality (CT, MRI, PET) was developed and subsequently applied clinically in human cancer patients, only then would it become available to veterinarians for clinical application. However, in the last two decades, the potential for companion species with spontaneously arising tumors to act as surrogates for preclinical development of advanced imaging technologies has become more apparent. Indeed, the blueprint has begun to shift where investigations of novel cancer imaging technologies in companion species precede human application and are involved earlier in the development pipeline. For example, the conformal image-guided radiation therapy technology, tomotherapy®, was first tested by inclusion of companion dogs with spontaneous sinonasal tumors and subsequent PET/CT serial imaging in these patients was used to characterize and map hypoxic, metabolic, and proliferative areas of the tumors under treatment (1, 2). Several other examples will follow in subsequent sections of this review.

### Opportunities, Advantages, and Obstacles for Comparative Cancer Imaging

The opportunities and potential advantages for a comparative approach to cancer investigation that involves the inclusion of companion species (e.g., pet dogs and cats) with spontaneously arising cancers as preclinical surrogates to human-centric studies have been collectively and generally discussed in several of the reviews within this special volume of Frontiers. With regards to cancer imaging in particular, the opportunities, and advantages more specifically center around issues related to body size (including spatial target/normal anatomic characteristics), physical size and spatial distribution of metastasis, tumor heterogeneity, the presence of an intact syngeneic immune system and a syngeneic tumor microenvironment shaped by the natural evolution of the cancer. Taken in totality, these comparative opportunities predict that safety, diagnostic, and efficacy data generated in companion species with naturally developing and progressing cancers would better recapitulate the human cancer condition than that of artificial models in small rodent systems.

The body size of companion species conveys several advantages in the imaging realm. Standard and advanced equipment designed for pediatric and adult human imaging of cancer can be readily applied without modification to companion species ensuring more global accessibility at both human and veterinary clinical research centers. Further, the use of similar equipment, hardware setup, software, and scan protocols provide the opportunity for standardization and harmonization of imaging operating procedures and quality assurance across imaging protocols, imaging hardware, and the imaged species. Spatial concerns regarding image resolution, radionuclide and other optically active compound wavelength and tissue penetration depth are also abrogated in companion species with larger body size. For example, when dealing with diagnostic and therapeutic radionuclides, the canine body size is more representative of human subjects than rodents with respect to radiation dosimetry calculations and are more reliable for extrapolation to humans. Murine models do not replicate the size and spatial distribution of human metastatic cancer and these factors strongly influence image resolution and dosimetry. This becomes even more important when investigating off-target effects of diagnostic and therapeutic radiation emitters where tumor and normal organ (e.g., bone marrow) proximity is critical to both efficacy and safety. Finally, body (and indeed tumor) size allows for serial and varied biospecimen procurement in companion species that are concomitant to image investigations. Since anesthesia is requisite for most advanced imaging modalities (CT, MRI, PET) in companion species, this allows greater ethical latitude for procurement of biospecimens concomitant to procuring advanced and functional images. Such procurements, whether they be from the peripheral blood compartment or the tumor/TME, would allow characterization of PK/PD for novel imaging agents, validation of potential imaging biomarkers, assessment of immune modulation, and safety evaluations.

Companion animals in general allow for achieving much higher quantitative imaging accuracy. Imaging under anesthesia allows much more reproducible positioning of companion animals within scanners and generally experience reduced motion artifact, very important in multiple sequential (treatment response assessment) types of studies. Similarly, anesthesia allows for prolonged scanning times, if necessary, therefore allowing more accurate assessment of PK (e.g., in dynamic PET studies of novel imaging or labeled treatment agents).

With the realization that tumor—tumor microenvironment (TME) interaction is critical for many aspects of tumor imaging, image-guided therapy, and image derived assessment of therapeutic response, the potential of syngeneic natural tumors in companion animals as surrogates for image technology development becomes self-evident. Further, an intact syngeneic immune system is a critical component of this tumor-TME interaction and further enhances the surrogate utility of cancer-bearing companion species in a comparative approach.

The following review will discuss the utility of comparative cancer imaging in more detail while providing several illustrative examples of investigational approaches performed or currently under exploration at our institutions. It is our hope that the examples presented should serve to provide those in cancer investigations who are unfamiliar with available comparative methodologies an understanding of the potential utility of this approach.

### NCI Perspectives

In 2004, the intramural research program of the National Institutes of Health's National Cancer Institute created the Comparative Oncology Program (COP), with the explicit goal of advancing the tumor-bearing dog as a naturally-occurring complementary animal model of cancer. As a scientific discipline, comparative oncology has the overarching goal to advance knowledge of veterinary cancers and to rationally integrate such patients into studies of cancer biology and therapy. To that end, in 2015, a workshop was convened by the U.S. National Academy of Medicine's National Cancer Policy Forum to define and explore the perceived and/or acknowledged gaps in knowledge that may impact the delivery of comparative oncology data to stakeholders in cancer drug development (3). Seven distinct topics, including opportunities in comparative cancer imaging, were discussed to gain insight into how the gaps could be addressed and the role of tumor-bearing dogs in cancer research strengthened. Since that time, several NCI-funded initiatives focused on canine cancer have been introduced to the cancer research community that begin to address these gaps in knowledge, including genomic characterization of canine tumors and the development of novel immunotherapeutic strategies.

As part of the U01-affiliated Immuno-Oncology Translational Network (https://www.cancer.gov/research/key-initiatives/moonshot-cancer-initiative/implementation/adult-immunotherapy-network) several exciting collaborative efforts are underway to realize the potential for dogs to inform the development and prioritization of novel immunotherapeutic approaches for human cancer treatment and many involve sophisticated imaging techniques (discussed later in this review).

### Consortia [COTC, CBTC, CORC]

One of the most important achievements in comparative oncology over the last decade has been the development of successful and collaborative consortia that perform multicenter clinical trials in the comparative realm, several involving development of, or utilization of advanced cancer imaging. Consortium infrastructures allow larger scale clinical trials and provide the voice for collective advocacy in veterinary and comparative oncology. Examples include the comparative oncology trials consortia (COTC), comparative brain tumor consortia (CBTC) and the comparative oncology research consortia (CORC).

The COTC is an active network of 24 academic comparative oncology centers (https://ccr.cancer.gov/Comparative-Oncology-Program/sponsors/consortium), centrally managed by the National Institutes of Health–NCI's COP that functions to design and execute clinical trials in dogs with cancer to assess novel therapies (4–6). The goal of this effort is to answer biologic questions geared to inform the development path of these agents for use in human cancer patients. COTC trials are pharmacokinetically and pharmacodynamically rich, with the product of this work directly integrated into the design of human early and late phase clinical trials. They are focused to answer mechanistic questions and define dose-toxicity and dose-response relationships. They can be designed to compare varying schedules and routes of drug administration, validate target biology, model clinical standard operating procedures (SOPs), and assess biomarkers. Additionally, within this effort, the COTC PD Core was created. The COTC PD Core is a virtual laboratory of assays and services, including pathology, immunohistochemistry, immunocytochemistry, flow cytometry, genomics, proteomics, cell culture and drug screening, PKs, and cell biology designed to support COTC clinical trial biologic endpoints. As of 2019, the COTC has completed 14 clinical trials and has been successful in promoting the utility of comparative oncology modeling within the drug development community.

The CBTC was created by the NCI's COP in 2015 to specifically address gaps in knowledge that pertain to the translational relevance of canine brain tumors to their human counterparts (https://ccr.cancer.gov/comparative-oncology-program/research/cbtc). Further, the CBTC membership seeks to recognize the potential for these canine patients to participate in studies of cancer biology and therapy to benefit both humans and dogs. At the inaugural meeting of this consortium, a SWOT analysis (strengths, weaknesses, opportunities, threat) was carried out to define, through working group discussions, a list of prioritized projects that could begin to address the most critical unanswered questions and medical needs for both species (7). Since that time, a series of initiatives have been published that reflect the activity of this group of investigators. These include a comparative assessment of canine glioma pathology and harmonized magnetic resonance imaging (MRI) parameters to facilitate multicenter clinical trials in canine brain tumor patients (8, 9). Further, the first CBTC-specific clinical trial in canine brain tumors evaluates a theranostic strategy targeting apoptosis. In this ongoing trial, dogs with meningioma receive a novel CNS-penetrant small molecule activator of procaspsase-3 (PAC-1) and undergo serial PET imaging with a novel apoptosis-specific PET imaging agent (^18^F-CSNAT4) to detect and semi-quantitatively measure tumoral apoptosis prior to and after PAC-1 exposure (10–12). Data generated from this work is directly linked to and is informing the clinical study of both PAC-1 and ^18^F-CSNAT4 in human patients (clinicaltrials.gov identifier NCT02355535).

The CORC is a recently formed research consortia whose members represent academic comparative oncologists and scientists from partnered academic institutional programs where an NCI Comprehensive Cancer Center and academic veterinary oncology program have a formal affiliation. The CORC was designed to fulfill some of the clear mandates required to advance the discipline of comparative oncology. The V Foundation for Cancer Research committed to serve as the fiduciary agent, funding partner, and grant coordinator of CORC in order to support fundamental and translational research to more fully characterize cross-species opportunities. (https://www.v.org/research/specialfunds/canine-comparative-oncology/). Advanced cancer imaging in companion species comprise an important component of this consortia.

## Imaging Biomarkers in Comparative Oncology

### Quality Assurance, Standardization, and Harmonization of Imaging Technologies

Until recently, the primary goal for imaging has been diagnosis and staging, both in humans and companion animals. However, when imaging is used to define a treatment target (e.g., in radiation oncology), or assess changes from one scan to another scan to assess treatment efficacy (e.g., by RECIST evaluation), imaging signals need to be *quantified*. Quantification requirements are elevated in both, spatial quantification information (e.g., where exactly is tumor), as well as temporal quantification (e.g., how much has the tumor changed). Because of the high spatio-temporal quantification nature, quantification imaging requirements are much more stringent; they require a high level of image quantification and minimal uncertainties of the imaging signal.

In order to obtain quantitative information from any images, several steps of the so-called “imaging chain” need to be followed. The key steps of the quantitative imaging chain include: (1) Imaging protocol, (2) Imaging data acquisition, (3) Image reconstruction, (4) Image analysis, and (5) Image measurement. In order to secure a high level of quantitative imaging accuracy, each step of the quantitative imaging chain needs to be carefully evaluated. As the overall image quantification accuracy depends on each step, it is essential to adequately control major uncertainties of the overall chain.

Because of the similarities of the imaging systems between human and larger companion animals, the same Quality Assurance (QA) steps as in humans should be followed in veterinary clinics [e.g., see Table 1 in Jeraj et al. (13)]. For quantitative imaging applications, the QA program needs to be even more elaborate (often requiring added scanner qualification using dedicated imaging phantoms), to minimize imaging bias and variance. In multi-center setting, the need for adequate QA at each participating site, is significantly increased. Furthermore, steps to ensure better “harmonization” of the scanners from multiple institutions (tuning them in a way to produce similar imaging quality), is warranted. While basic imaging QA is typically performed on scanners in veterinary use, a shortage of adequate expertise, particularly medical physics, which assures high quality imaging for human use, severely hampers ability to perform quantitative imaging in comparative oncology setting.

In order to help facilitating quantitative imaging, the Quantitative Imaging Biomarker Alliance (QIBA) has been established (https://www.rsna.org/en/research/quantitative-imaging-biomarkers-alliance). QIBA seeks to improve the value and practicality of Quantitative Imaging Biomarkers (QIBs) by reducing variability across devices, sites, patients and time (14). QIBA defines a QIB as “an objective characteristic derived from an *in vivo* image measured on a ratio or interval scale as indicators of normal biological processes, pathogenic processes, or a response to a therapeutic intervention.” The technical performance of each QIB must then be assessed by establishing the physical phenomenon being measured, under what circumstances it can be measured, and what level of uncertainty is to be expected with each measurement. These points are all addressed by investigating the *bias, repeatability*, and *reproducibility* of measured imaging values.

While in humans, quantitative imaging has been rather well-established, realization of the needs and requirements for achieving adequate quantitative imaging accuracy in comparative oncology is significantly lagging behind. Too often imaging is used without adequate quantitative accuracy, and without understanding uncertainties, which can severely hamper interpretation of the imaging data. For example, QIBA has developed a number of “profiles” and “protocols (http://qibawiki.rsna.org/index.php/Profiles), in various stages of validation, which address necessary steps that need to be taken for achieving adequate image quality for a given type of imaging accuracy (e.g., FDG PET/CT). Ideally, one would derive similar “profiles” for companion animals. In the meantime, it is highly recommended that QIBA recommendations are also followed in comparative oncology studies.

## Utility of Comparative Cancer Imaging

### Imaging Technology Development

#### Contrast Agent Development

**Figure 1:**
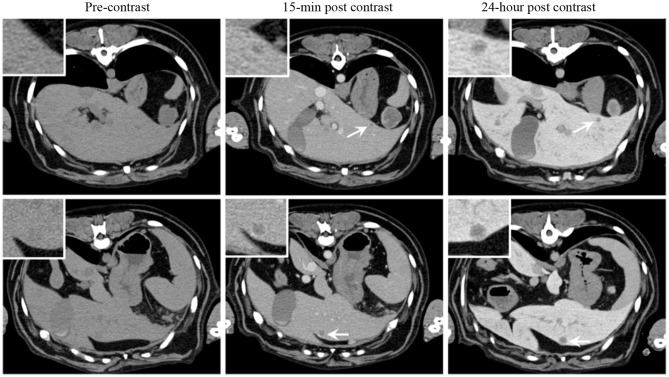
Signal enhancement pattern of a long-circulating liposomal iodinated CT contrast agent investigated in a companion dog with metastatic liver tumors. Axial CT images demonstrating the effect of post-Liposomal-I imaging time point on visualization of metastatic liver lesions (white arrows). The 0.5 cm **(Top)** and 1 cm **(Bottom)** lesions are better visualized on the post-24 h images due to increased liver uptake of the contrast agent. Reprinted from Ghaghada et al. (15).

Owing to the similarities in size, spatial anatomy and physiologic parameters (e.g., ADME; absorption, distribution, metabolism, and excretion), cancer-bearing companion species enable proof-of-concept investigations of novel imaging agents with relevant lesion sizes on clinically-equivalent scanners. A recent investigation of a long-circulating liposomal iodinated CT contrast agent in dogs with naturally occurring tumors serves illustrative of this concept (15). Unlike conventional iodinated contrast agents which have rapid wash-in/wash-out tumor kinetics and renal clearance, long-circulating liposomes gradually extravasate, through the permeable tumor vasculature, and accumulate in tumors, a phenomenon known as enhanced permeation and retention (EPR) (16). Our trial in companion dogs characterized agent safety and ability to perform serial and prolonged visualization of small and large tumors over time without repeated infusions as is necessary in non-liposomal iodinated contrast agents. The agent allowed significant enhancement and uniform opacification of the vascular compartment in early-phase scans (15-min post-contrast infusion), demonstrated non-renal clearance which is preferable in patients with renal impairment, and took advantage of EPR characteristics of long-circulating liposomal products allowing intra-tumoral signal enhancement in the delayed-phase scans (24 h post-contrast infusion) (Figure 1).

#### Novel Radionuclide Tracer Development

**Figure 2:**
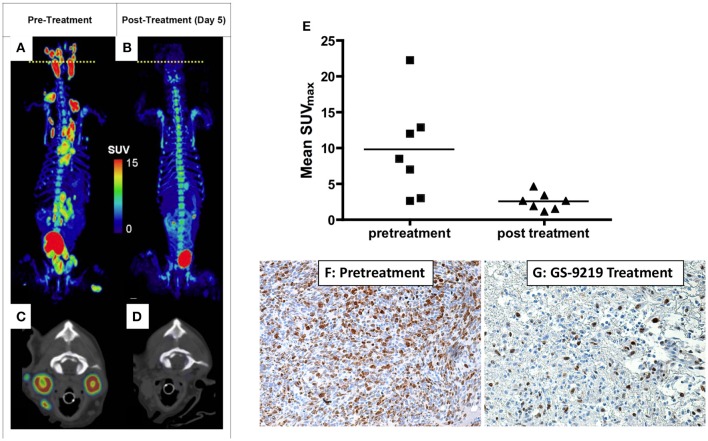
PET/CT scan before **(A,C)** and 5 days after **(B,D)** a single dose of GS-9219 in a dog with NHL. The cell proliferation tracer ^18^F-FLT was used to document significant anti-proliferative response in affected lymphoid tissues (popliteal, mesenteric, mediastinal, prescapular, and submandibular lymph nodes). Note that the signal in the urinary bladder and renal calyces is normal and represents urinary excretion. Low-level signal present in the vertebral bone marrow and the gastrointestinal tract reflects background uptake of tracer in the proliferating cell populations in these tissues. The axial views are displayed at the position of the yellow dotted lines on the whole-body PET scans and represent the level of the mandibular lymph nodes. **(E)** Mean FLT maximum body mass standardized uptake value (SUV_max_) for seven dogs was significantly higher before treatment than after treatment. Mean FLT SUV_max_ predicted a significant decrease in tumor proliferation as confirmed using Ki-67 immunoreactivity as illustrated in **(F,G)**: Lymphoma tissue from the prescapular lymph node of a dog before treatment **(F)** compared with the contralateral prescapular lymph node in the same dog biopsied 4 days following treatment **(G)** (X600). Note the significant decrease in Ki-67 immunoreactivity following therapy indicating an antiproliferative effect. **(A–D)** Reprinted from Reiser et al. (17). **(E–G)** reprinted from Lawrence et al. (18).

**Figure 3:**
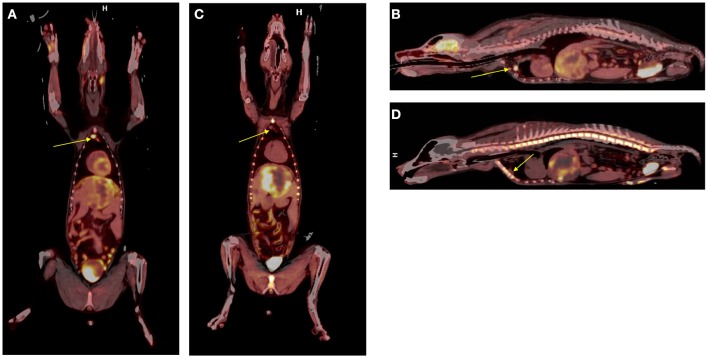
Dual tracer ^18^F-FDG **(A,B)** vs. ^18^F-FLT **(C,D)** in canine hepatocellular carcinoma. Note the increased tumor conspicuity with ^18^F-FLT particularly when discerning tumor borders from surrounding hepatic parenchyma. A reactive retrosternal lymph node is evident within the ^18^FDG images (arrows, **A,B**) but does not exhibit significant ^18^FLT uptake (arrows, **C,D**).

The opportunities and advantages of the comparative approach also lends itself to development and validation of novel radionuclide tracers for functional imaging. In particular, spatial (lesion-normal tissue) considerations and our ability to perform serial biopsy and biospecimen procurement allow concomitant validation of novel tracer functional correlation. For example, the authors validated the non-invasive assessment of tumor proliferation of the thymidine-analog 3′-deoxy-3′[^18^F]fluorothymidine (FLT) by comparing FLT uptake in companion dogs with non-Hodgkin's lymphoma (NHL) to gold-standard Ki-67 immunohistochemistry [Figure 2; (18)]. Comparisons of ^18^F-FDG and ^18^F-FLT may also allow distinction of tumor borders within areas of higher-than-average background tracer uptake, such as within liver or brain (Figure 3). Further, these studies illustrate the use of advanced imaging in companion species to document efficacy of investigational cytotoxic agents like the novel cytotoxic prodrug, GS-9219; and also to map areas of chemosensitive cells such as proliferating bone marrow (17, 19, 20). Access to such patients supports multiparametric imaging studies in a tractable period of time within their clinical management.

**Figure 4:**
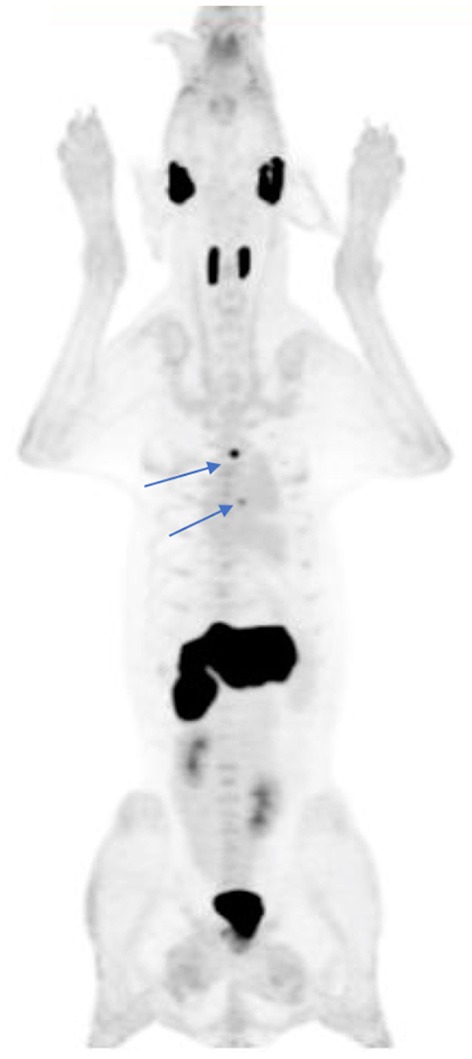
^18^F-tetrafluoroborate, a PET imaging agent for the sodium-iodide symporter (NIS) protein, was used to document physiologic distribution in this 41 kg hound dog. Note the expected physiologic uptake within the salivary glands, thyroid, and stomach. Small foci of uptake in the thorax (arrows) represent ectopic thyroid tissue within the left ventricular outflow tract and mediastinum. There is evidence of tracer excretion within the urinary system (renal pelvis and bladder). A small amount of free F-, the product of tracer dehalogenation *in vivo*, is evident by the mild diffuse bone uptake in this MIP image.

**Figure 5:**
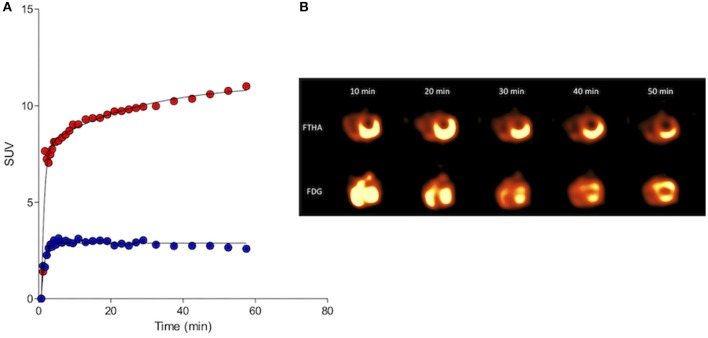
Kinetic standardized uptake value (SUV) data (**A**; Red, 18FDG and blue, 18FTHA) and serial left ventricular images **(B)** gathered from the myocardium of a clinically normal purpose-bred cat. Both 18FDG and 18FTHA were evaluated 48 h apart, with DICOM data collected over a 1 h period beginning simultaneously with tracer injection. Note the early trapping and sustained retention of 18FTHA contrasting with continuously increasing uptake of 18FDG over the 60 min imaging period. This is consistent with the continuously gluconeogenic and glycolytic state of the domestic cat and its myocardium, respectively, even in the fasted state.

Other examples of novel tracer development and application by inclusion of companion species include 13-C pyruvate magnetic resonance spectroscopy (21), and ^18^F-tetrafluoroborate (^18^${\text{F-BF}}_{4}^{-}$ or ^18^F-TFB) for expressed sodium-iodide symporter (NIS) PET imaging (Figure 4), which could be a useful tool for clinical thyroid and neuroendocrine tumor imaging, for preclinical imaging of NIS-expressing disease models and for cell trafficking studies (22–24). We have also explored the utility of an ^18^F-radiolabeled fatty acid ^18^F-fluoro-thia-heptadecandoic acid for metabolic imaging of fatty acid oxidation, which is an emerging field in the study of cancer metabolism (Figure 5) (25, 26). All of these concepts share the common theme of investigating advanced functional imaging technologies in a relevant large animal model that can develop spontaneous syngeneic tumors.

### Assessment of Novel Cytotoxic Drug Efficacy

Inclusion of companion species in the pre-clinical assessments of efficacy and safety of novel cytotoxic drugs holds tremendous potential for informing subsequent or parallel human clinical trials and theoretically could accelerate the drug development and registration timeline. The inclusion of advanced cancer imaging in these comparative trials serves to provide compelling proof-of-concept characterizations of response in small treatment cohorts. Further, characterization of early imaging response may then be followed and validated as a predictive measure of temporal response such as progression-free and overall survival. As illustrated above in Figure 2, inclusion of companion dogs in a proof-of-concept investigation of a novel cytotoxic nucleotide analog prodrug (GS-9219), response assessment using advanced cancer imaging (e.g., ^18^F-FLT PET/CT) could be performed early (within days of treatment) and was predictive of outcome (17–19). These studies also serve to highlight the potential bidirectional flow of comparative oncology advances; GS-9219 was eventually abandoned in the crowded human NHL therapeutic arena, but continued development in the veterinary arena and represents the first FDA-approved cytotoxic chemotherapeutic for use in pet dogs with lymphoma under the name Tanovea® (27–29).

**Figure 6:**
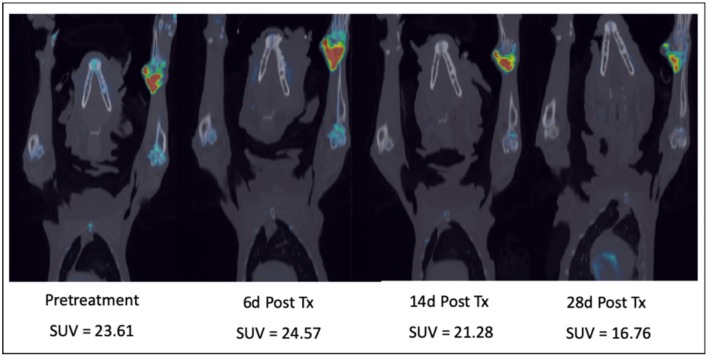
Serial combination ^18^F-NaF/^18^F-FLT PET/CT scans in a dog with a right distal radial osteosarcoma treated with a novel bone-targeting bisphosphonate-cytotoxic drug conjugate. After an initial proliferative increase 6 days after a single IV drug treatment, tumor proliferation and bone turnover diminished over the 28-day treatment cycle. SUV, Maximum Standard Unit of Value.

As a second example, our group has used a comparative approach to use advanced imaging to temporally characterize the effects of a novel bisphosphonate-cytotoxic drug conjugate that targets primary or metastatic sites of cancer within bone (30). Prior to first-in-human trials we performed a proof-of-concept pilot trial of safety and efficacy in companion dogs with spontaneously arising osteosarcoma and applied serial ^18^F-NaF/^18^F-FLT PET/CT cancer imaging to assess primary tumor and associated bone proliferative activity [Figure 6; (31)]. The novel agent was found to induce an initial increase in proliferative activity at the tumor location at day 6 post-treatment followed by reduced primary tumor proliferation over the course of the next 28 days. These data were correlated with documentation of pain palliation using force-platform analysis and significant suppression of the bone resorption marker urinary NTX-telopeptide (31). These results, along with adverse event profiling in companion dogs, justified further clinical advancement of MBC-11, and subsequent first-in-human investigations documented substantial reductions in metabolic activity of several solid bone cancers in human patients at well-tolerated doses (32).

Another example that highlights the utility of molecular imaging in gauging tumor response is an investigation of serial ^18^F-FDG PET/CT during toceranib treatment in dogs with solid tumors (33). This study exemplifies the potential for discordance with the use of ^18^F-FDG PET to gauge response to receptor tyrosine kinase inhibition and the need to consider the timing of such imaging studies, as well as applying multi-parametric imaging-based measures of response such as dual ^18^F-FDG and ^18^F-FLT PET/CT imaging, to gain a clearer understanding of drug impact. In the context of short drug exposures, an immediate anti-proliferative response (best assessed through ^18^F-FLT PET imaging) may predate a metabolic response (as assessed through ^18^F-FDG PET imaging), as suggested in a study of human patients receiving sunitinib treatment for renal cell carcinoma (34). Additionally, issues relating to structural changes within tumors, such as necrosis and hemorrhage, may have impacted the uptake of ^18^F-FDG in the canine patients receiving toceranib thus leading to discordant results. It is also important to consider the clinical benefit patients may experience during therapy that may not be captured with standard treatment response assessment criteria (e.g., RECIST or PERCIST) alone. Nonetheless, the examples highlighted here serve to illustrate the utility of comparative cancer imaging within the drug development pathway for novel cytotoxic therapeutics, as well as the opportunities to explore various schedules for gathering imaging data to determine which are most informative.

### Assessment of Tumor Microenvironment (TME)

Solid tumors are phenotypically heterogeneous, exhibiting an array of expressed characteristics that are rooted both in their origin and tumor microenvironment. Extracellular changes alter the tumor microenvironment in a manner that can cause further modifications of genes and their transcription at the epigenetic level. Temporal evolution of these heterogeneities is affected by the interplay between the dynamics of the tumor, tumor microenvironment, neovasculature, and any therapeutics administered, all of which elicit responses in the form of further genetic and phenotypic modifications. It is no surprise that assessment of tumor microenvironment has been of specifically high interest to investigators both in human and comparative oncology.

One of particularly impactful tumor microenvironment characteristics that has shown to have great influence over clinical outcome and response to therapy is hypoxia (35, 36). Non-invasive volumetric imaging of hypoxia markers continues to become a more ubiquitous technique for *in vivo* visualization and quantification. The most common imaging modality, also quite frequently used in comparative oncology setting, is PET using specific radiotracer surrogates of tumor hypoxia, such as ^18^F-FMISO, ^18^F-FAZA, and ^61,64^Cu-Cu-ATSM, among others. FMISO is the most common PET hypoxia tracer due to its close chemical relationship with the marker pimonidazole. Low image contrast of FMISO was addressed with the synthesis of FAZA, a less lipophilic nitroimidazole that is not plagued by non-specific uptake in normal tissues. Similarly, Cu-ATSM lipophilicity from planar molecular geometry allows for rapid passive uptake in tumor cells, where it is preferentially reduced by cytochrome reductase enzymes forming the microsomal electron transport chain that leads to high image contrast.

Similarly, functional MRI can be used to indirectly image hypoxia. The BOLD technique in particular images the differential blood flow and the oxidation of hemoglobin via changes in magnetic susceptibility. Dynamic contrast-enhanced MRI (DCE-MRI) allows for assessment of vasculature parameters (e.g., perfusion/permeability), related to tumor microenvironment, and indirectly to tumor hypoxia, some of which has been shown to be predictive of outcome both in humans as well as in canines (37).

Imaging of tumor microenvironment, together with imaging of other clinically relevant tumor phenotypes such as proliferation, metabolism, and vasculature condition assessment, has shown to be of significant benefit to comparative oncology, as it allows better understanding of interplay between different tumor phenotypes, assessment of the response to therapeutic interventions, and ultimately deriving better and more effective treatments (2, 38–40). However, more research, and particularly broader adaption of advanced imaging technologies is needed to fully explore its potential.

### Response Prediction and Assessment

#### Functional Assessments

The utility of advanced imaging to serially assess functional changes in tumors or TME to document or indeed predict response to cancer therapeutics has been covered in previous sections of this review [see sections Imaging Technology Development, Assessment of Novel Cytotoxic Drug Efficacy, and Assessment of tumor microenvironment (TME)]. As the field of comparative oncology continues to advance and novel interventions and therapeutic strategies are investigated in companion species, the integration of advanced imaging technologies to document response and develop non-invasive image-based biomarkers will become more important and commonplace.

#### Immunotherapeutic Response Assessment

Although immunotherapy is becoming one of the cornerstones of modern cancer therapy, resulting in durable favorable outcomes for some patients, the assessment of clinical response to immunotherapy is still a very challenging task (41). Immunotherapy response patterns can be substantially different from those of classical cytotoxic therapies (42). A significant subset of patients first experience a pseudo-progression after the administration of immunotherapy, and the actual response/shrinkage of tumors can be delayed and only observed later in the time course of therapy. Four different distinct immunotherapy response patterns are associated with favorable survival (42); (a) shrinkage in baseline lesions with no new lesions, (b) long-term stable disease, (c) response after an initial increase (pseudoprogression) of tumors, and (d) response of initial lesions but with appearance of new lesions. Therefore, the standard Response Evaluation Criteria in Solid Tumors (RECIST 1.1.) (43) are not appropriate for assessing the effects of immunotherapy and can result in patients moving off of effective therapy due to an invalid parameter for progression. For example, as defined by RECIST, patients experiencing early pseudo-progression or patients with response in the presence of new lesions would be characterized as progressive disease (PD), indicating treatment failure and suggesting cessation of therapy. However, such treatment response patterns following immunotherapy can be associated with eventual tumor regression/stabilization and potentially long-term survival. In 2009, immune-related response criteria (irRC), based upon data from checkpoint blockade were recommended for use in immunotherapy (42). irRC is based on measuring the change in size of tumor burden and the change in the number of metastatic lesions at two different imaging time points, at least 4 weeks apart. While the irRC have been retrospectively validated, their usefulness and generalizability to other immunotherapy agents and cancer types is still undergoing prospective validation (42, 44–50). Broadly speaking, irRC only covers assessment of anatomical changes, which are known to be slow, compared to molecular and functional changes within the tumor and tumor microenvironment, and has been inconsistently implemented (45, 46, 49, 50).

Non-invasive imaging tools to assess and predict response to immunotherapy would greatly facilitate drug development and clinical decision-making in this era of growing incorporation of immune-modulating approaches for treatment. Ideally, methods that assess response to immunotherapeutic strategies as early as possible should allow non-responding patients to switch to other treatment modalities sooner and guard from the high cost and toxicities of continuing an ineffective therapy. Functional/molecular imaging is known to show tumor changes much earlier than anatomical changes. While ^18^F-FDG PET/CT has become a standard tool to assess treatment response in oncology, in immunotherapy its interpretation is severely confounded by changes related to an active and responding immune response. The lack of specificity of FDG uptake (e.g., Figure 3) mandates a more specific indicator of tumor cell viability, which fortunately can be achieved with ^18^F-FLT PET/CT or PET/MR imaging. We are currently exploring in companion species whether a positive immunotherapeutic response will be reflected by an increased FDG/FLT uptake ratio [termed the Imaging Immune Response (IMR) ratio] during and after therapy when compared with baseline. An increase in the IMR ratio is expected because of two premises: (1) effective immunotherapy elicits immune activation leading to increased “inflammation” in the tumor reflected by increased glycolytic activity in the tumor microenvironment by activated immune cells, which can be measured by FDG PET, and (2) effective immunotherapy will eventually result in antitumor effects, which can be measured by a decrease in proliferation as reflected by thymidine synthase activity in the tumor region measured using FLT PET. As antitumor effects can be delayed, and early changes on FDG PET/CT may reflect both inflammatory and tumor progression, we hypothesize that the change in the ratio of FDG to FLT will provide the necessary discriminatory information to characterize tumor lesions as having a *positive immune effect* (increase in IMR ratio) vs. *no effect* (no change or decrease in IMR ratio).

The use of imaging as a predictive biomarker is currently limited due to the contrasting reports and limited evidence about the predictive power of simple standardly applied PET metrics, such as the maximum standardized uptake value (SUV_max_) (51–55). On the other hand, the rapidly expanding field of medical image analysis, so called “radiomics,” is harnessing the full power of medical imaging by extracting numerous quantitative features (sometimes referred to as “texture features”) out of the images of different modalities, including PET, CT and magnetic resonance imaging (MRI) (56). Several studies have reported predictive ability of radiomics texture features in different types of cancer therapies, but interestingly, no radiomics analyses have been performed in immunotherapy studies thus far.

Applied to immunotherapy, one would expect that the immune-radiomics (irRADIOMICS) signature of responders will be different from the irRADIOMIC signature of non-responders due to the different levels, spatial distribution and temporal dynamics of tumor infiltrating lymphocytes (TILs) and various other immunosuppressive cells, such as myeloid-derived suppressive cells (MDSC), regulatory T cells (Treg), tumor-associated macrophages (TAM), and regulatory dendritic cells (DCreg) (57). Thus, it may be possible to assess the response to immunotherapy much earlier than by conventional means, perhaps even at just one imaging time-point, preferably in the pseudoprogression phase. Based on the assumption that irRADIOMICS might be able to detect differences in the tumor immunosuppressive microenvironment, we further hypothesize that it may also be possible to predict which patients are most likely to benefit from immunotherapy before initiation of the therapy. In other words, irRADIOMICS could have potential to serve as a pre-treatment biomarker of response to immunotherapeutic strategies.

**Figure 7:**
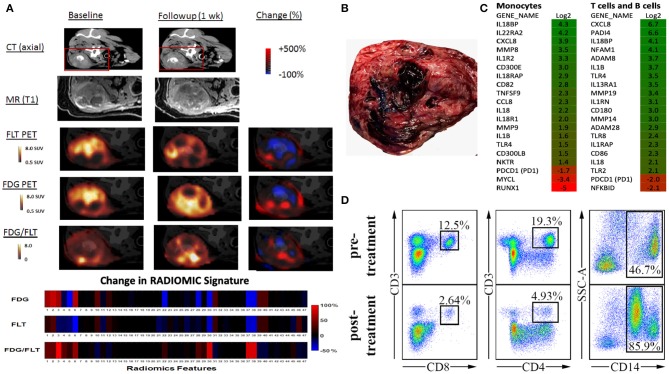
Advanced imaging (irRADIOMICS) evaluation in a dog with osteosarcoma before and after immunotherapy and correlation with biomarkers of immunomodulation. **(A)**. CT, MRI, ^18^F-FLT PET, ^18^F-FDG PET, and FDG/FLT PET images of a dog with a stump recurrence osteosarcoma at baseline and 1 week post-L-MTP-PE immunotherapy. The change in image features using 47 extracted imaging features (RADIOMIC signature) extracted from ^18^F-FLT PET to ^18^F-FDG PET and the ratio of the FDG/FLT PET scans are presented in the lower bars. Analysis shows that the change in FDG PET radiomics signature is mostly positive, FLT PET radiomics signature mostly negative, and change in FDG/FLT PET ratio radiomics signature is uniformly positive, likely indicating effective immunotherapy. The RADIOMIC features can then be correlated with global tumor pathology/histology **(B)** and other biomarkers of immunomodulation such as single-cell gene expression profiling **(C)** and peripheral blood mononuclear cell subset **(D)** changes from baseline.

**Figure 8:**
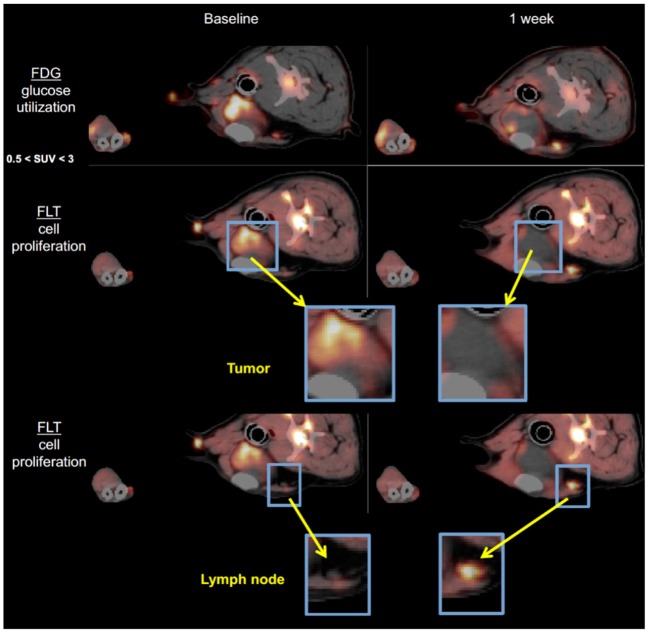
^18^F-FDG PET and ^18^F-FLT-PET images of a dog with metastatic melanoma before and after immunotherapy with intratumoral immunocytokine injections. Note the antiproliferative response in the index tumor and the conversely pro-proliferative response in the non-metastatic, reactive regional lymph node predicting a positive antitumor immune response.

To begin characterizing response assessment and predictive potential, we are currently creating irRADIOMIC signatures in companion dogs with metastatic osteosarcoma (Figure 7) and melanoma (Figure 8) before and after immunotherapeutic strategies. Figure 7 represents a veterinary patient who developed a stump-recurrence osteosarcoma following forelimb amputation and platinum-based chemotherapy. The dog received systemically delivered liposomal muramyl tripeptide (L-MTP), an innate immune system stimulant known to have activity in canine and human osteosarcoma and to activate canine monocytes (58–60). In order to assess metabolic and proliferative response of this dogs tumors (and regional lymph nodes), and account for potential changes in perfusion/permeability, we then performed dynamic ^18^F-FLT PET/CT and dynamic ^18^F-FDG PET/MR scans before and 1 week after initiation of immunotherapy. Analysis revealed that the change in FDG PET radiomic signature was mostly positive, FLT PET radiomics signature mostly negative, and the change in FDG/FLT PET ratio (IMR ratio) radiomic signature was uniformly positive, suggesting an effective immune response. Figure 8 represents a veterinary patient with a large prescapular metastatic malignant melanoma from a subungual primary who received intratumoral injections of an investigational immunocytokine-monoclonal antibody fusion protein. Pre and post-treatment FLT and FDG PET/CT images revealed reduced metabolism and proliferation in the prescapular metastatic lesion with increased metabolism and proliferation in the non-effaced reactive regional lymph node; also indicative of an effective immune response.

Through serial biospecimen procurement during serial scans, we have begun to correlate radiomic signatures with histologic and immunopathologic characterizations of the tumor, the tumor microenvironment, peripheral blood compartment and regional nodes. Our group, as part of the Moonshot U01-affiliated Immuno-Oncology Translational Network (IOTN: https://www.cancer.gov/research/key-initiatives/moonshot-cancer-initiative/implementation/adult-immunotherapy-network) has access to an ever-expanding catalog of validated canine-specific reagents and methodologies that will allow assessments that should prove meaningful and readily translatable. We have demonstrated an ability to apply sophisticated analytic interrogations in dogs with solid tumors and have demonstrated differences before and after a variety of immunomodulatory therapies. For example, in the dogs with metastatic OSA receiving immunotherapy (Figure 7), we assessed changes in PBMC lymphocyte subsets, flow-sorted immune cells, and documented changes in gene expression for known activation pathways, and optimized a protocol for deep sequencing the canine T cell receptor (TCR) from PBMCs and showed increased TCR diversity after immunotherapy. These biospecimen-heavy pilot studies again illustrate the potential for the comparative approach to cancer image-based investigations. Much remains to be learned about standardization of irRADIOMICS imaging applications and analysis. Which textures are most critical to assess and the temporal nature of the signature changes that occur, are currently unknown and this knowledge is critical to determine the optimal timing of imaging events relative to the initiation of immunotherapy.

### Development of Novel Theranostic Approaches

Much utility is gained in clinical practice if a single agent can be used for both diagnostic imaging and therapy; so called “theranostic” agents. For example, metaiodobenzylguanidine (mIBG) is such an agent for pediatric neuroblastoma where ^123^I-mIBG is used for accurate staging and ^131^I-mIBG is the correlate therapeutic (61). Similarly, ^177^Lu prostate-specific membrane antigen (^177^Lu-PSMA) is used as a theranostic for men with prostate cancer (62).

The inclusion of companion animals in the development of novel theranostic agents also has advantages owing to their physical size and spatial distribution of tumors (primary and metastatic) which more closely mimics that in humans with cancer. This is critical for studying the safety and efficacy of theranostic agents that deliver therapeutic agents in close proximity to organs at risk, particularly lymphoid organs (bone marrow, spleen, thymus, draining lymphatics). This is particularly requisite for theranostic agents used for molecularly targeted radionuclide therapy (MTRT). Dosimetry calculations using canines should be more reliable for extrapolation to humans than mouse models.

**Figure 9:**
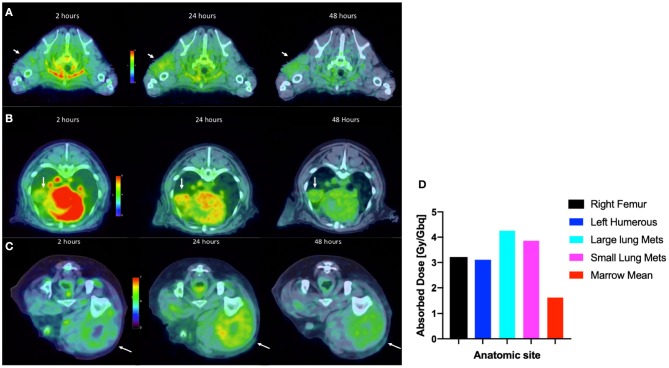
Tumor selective uptake of ^86^Y-NM600 as documented by PET/CT. 2-, 24-, and 48-h axial scans at the level of **(A)** left proximal humoral subcutaneous osteosarcoma metastasis (arrow); **(B)** left middle lung lobe metastasis (arrow); **(C)** right lateral femoral subcutaneous metastasis (arrow). Note the NM600 is primarily in the vascular compartment at the 20 h time point, then selectively disperses into all metastatic sites at subsequent time points. **(D)** Importantly, metastatic lesions had at least a 2:1 tumor to bone marrow uptake of ^86^Y-NM600 predicting safe delivery of ^90^Y-NM600.

By way of illustration, our group is working with radiolabeled alkylphosphocholines (APCs) which selectively accumulate in tumor cells *in vivo* by exploiting the relative overabundance of lipid rafts in cancer vs. normal cells, a mechanism that is ubiquitous to most malignancies (63, 64). An APC analog, NM600, developed by members in our group targets numerous cancer types regardless of histology and anatomic location (63). NM600 chelates a variety of radiometals (e.g., ^86^Y, ^90^Y, ^177^Lu, ^225^Ac) and is currently being evaluated in multiple imaging/therapy trials. Members of our collaborative group have eloquently shown that distant metastatic sites serve as a nidus for immunosuppressive cells (e.g., Tregs), and these mediate systemic immunosuppressive effects that antagonize external beam radiation therapy (EBRT) generated *in situ* vaccination—a phenomenon called concomitant immune tolerance (CIT) (65). Fortunately, CIT is radiation sensitive; delivering low-dose (~2 Gy) RT to metastatic sites can overcome CIT and enable *in situ* vaccine regimens to destroy both primary and distant tumor. (66). While it is not typically feasible to deliver EBRT to all sites of metastatic disease (due to immune suppression and inability to specifically target all microscopic disease), it may be possible to use MTRT to immunomodulate the TME of all tumor sites in the setting of metastatic disease. We are currently investigating delivery of low dose molecularly targeted radionuclides to all tumor sites in the setting of metastatic disease by using the theranostic isotope pair, ^86^Y-NM600 and ^90^Y-NM600, to immunomodulate the collective TME in a way that will promote response to EBRT-based *in situ* vaccine. Radiolabeled NM600 enables tumor-specific PET imaging (^86^Y-NM600) and targeted delivery of ionizing radiation (^90^Y-NM600) at doses that theoretically will abrogate CIT. Figure 9 shows a veterinary patient with widespread metastatic osteosarcoma undergoing serial ^86^Y-NM600 PET/CT imaging at various metastatic sites. These data proved selective uptake of NM600 by all metastatic sites and allowed dosimetry calculations (Figure 9D) that predicted at least a 2:1 tumor to bone marrow differential uptake and safe delivery of ^90^Y-NM600 to all metastatic tumors at doses likely to overcome CIT while sparing bone marrow. Indeed, this patient subsequently received the calculated ^90^Y-NM600 dose without hematologic toxicity. This example further supports the utility of the companion animal model for bridging preliminary rodent data and clinical application in people.

Other examples of companion species utility in the development of theranostic approaches include the previously mentioned use of the novel apoptosis-specific PET imaging agent (^18^F-CSNAT4) to detect and semi-quantitatively measure tumoral apoptosis prior to and after anti-apoptosis therapy (10–12).

### Miscellaneous Utility of Comparative Imaging

#### Cellular Trafficking

With heightened interest for the inclusion of companion dogs with heterogenous spontaneous tumors occurring and progressing in the context of a syngeneic TME and an intact immune system, the ability to image, in real time, changes in tumor and TME infiltrating immune effector and suppressor cell trafficking would be highly advantageous to assess effectiveness of immunotherapeutic approaches and characterize cell-based immune approaches.

**Figure 10:**
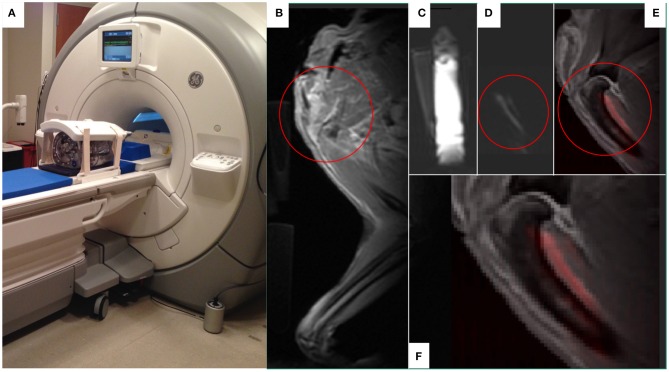
The use of ^19^F MR to develop *in vivo* imaging of ^19^F-labeled immune effector cell persistence and trafficking. A customized ^19^F MR coil system (MRI Tools, Berlin, Germany) that allows *in vivo* imaging of ^19^F. The duel-frequency, 8-channel Transmit/Receive ^1^H/^19^F torso coil system (MRI Tools, Berlin, Germany) with a water phantom is demonstrated **(A)** on a 3.0T Discovery MR750w MRI scanner (GE Healthcare, Chicago, IL). A companion dog limb was imaged using this system after ^19^F-emulsion was infused into the soft tissues on the caudal aspect of the proximal humerus, a common site for osteosarcoma. Fluorine images of the canine limb were acquired with a slice thickness of 10 mm via a T1-weighted gradient echo sequence (TR:20 ms, TE:2.1 ms, FA:20°, FOV: 380 × 380 mm, Matrix:128 × 128, resolution: 2.97 × 2.97 mm, number of averages: 64, *T* = 16 min). The MR proton anatomical image of the proximal humeral area of interest circled in red **(B)** were subsequently overlaid with the ^19^F images (**C**, fiducial marker; **D**, limb) to create a composite image **(E,F)** with ^19^F appearing as red in the composite images. Figures courtesy of Sean Fain and Paul Begovatz.

By way of example, we and others have been investigating the utility of natural killer cell (NK) based therapies in both companion dogs and pediatric patients with solid tumors (67, 68). Currently, there are no FDA-approved agents available to label and track immune cells after infusion into patients, and current infusion treatments are “blind” without confirmation that cells are viable or trafficking to tumor sites. Confirming delivery of cell therapies to tumors and other sites of disease will become more important as treatments are tailored to individual patients or modulated over time with repeated dosing. In clinical trials, we rely on analyses of blood and bone marrow samplings to detect the persistence of donor-derived infused NK cells; biopsy of tumors to actually localize these infused NK cells are difficult due to the potential of sampling error and risk to the pediatric patient. Thus, the field of cancer immunotherapy is in need of a means by which to non-invasively track infused cells in both normal organs and tumors. According to the FDA Cellular, Tissue and Gene Therapy Advisory Committee, there is an urgent need to track immune cells *in vivo* to determine trafficking patterns and longevity. Also, the FDA's Center for Devices and Radiological Health has launched an initiative to reduce unnecessary radiation exposure from medical imaging. MRI is the clinical standard for obtaining non-radioactive high-resolution images of soft tissue including solid tumors. While conventional MRI detects tissue ^1^H, and mainly differences in signal recovery of water and fat, multinuclear, and spectroscopic MRI has the potential to detect functional and cellular signals not visible with conventional ^1^H MRI methods. ^19^F MRI is a promising approach for tracking NK cells non-invasively without toxicity or ionizing radiation. Members of our collaborative group have developed methodology to label canine NK cells with non-radioactive ^19^F without compromising NK cell function and they were the first group to enumerate and track NK cells within a tumor *in vivo* using hot spot ^19^F-MRI (69, 70). We are currently evaluating the utility of a customized ^19^F MR coil (Figure 10) large enough to acquire images from canines (and pediatric patients in future trials). We have collected and expanded canine NK cells from University of Wisconsin Veterinary Care patients and have begun investigations to refine ^19^F-MR imaging protocols to characterize trafficking and persistence of autologous canine NK cells after intratumoral and intravenous infusion. Success of this line of investigation would offer a non-radioactive approach of tracking *ex vivo* activated NK cells (and other immune cells) in patients with solid tumors undergoing immune-based therapies.

As another example, one could envision the inclusion of companion dogs with cancer to bridge early investigations of dendritic cell migration in rodents using the novel PET probe ^18^F-tetrafluoroborate that was mentioned above (22–24).

#### Intraoperative Imaging

The use of advanced imaging modalities to inform surgical oncology decision making is another area where an opportunity for comparative oncologic investigations involving inclusion of companion animals with spontaneous tumors has and will play a role. Many of these technologies strive to discriminate between different tissue types (particularly between tumor and adjacent normal tissues) using intraoperative imaging. For example, the real-time intraoperative assessment of the completeness of surgical margins, sentinel node and distant regional metastasis at the time of tumor resection would be advantageous to waiting for time and labor intense *ex vivo* assessments and may abrogate the need for revision surgery. Examples where companion species have been involved in evaluating intra-operative surgical margins include optical coherence tomography (OCT) in dogs and cats with soft tissue sarcoma (STS) and fluorescence-guided surgical and sentinel node assessment for a variety of solid tumors (e.g., primary lung tumors, carcinomas, and STS) (71–78). Fluorescent probes used in these studies include agents with preferential/differential avidity for tumor cells such as protoporphyrins, lipid nanoparticles, integrin-targeting compounds (α_v_β_3_), folate-targeting agents, modified chlorotoxins these technologies have also been used to assess surgical wound beds for residual tumor cells after tumor extirpation (72).

## Summary and Future Directions

The myriad of opportunities and advantages inherent in the inclusion of cancer-bearing companion species in the investigation, development, and application of advanced imaging technologies stem primarily from similarities they share with cancer-bearing humans in body size, tumor heterogeneity, spatial distribution of metastatic disease, and the presence of an intact and syngeneic immune system and tumor microenvironment (TME).

The expansion of comparative consortia and funding opportunities that bring veterinary and physician-based basic researchers, medical physicists, and clinician-scientists to the cooperative table has the potential to harmonize the efforts applied in cancer imaging toward the common goal of diminishing the morbidity and mortality of cancer in all species. This bidirectional flow of new information should serve to streamline, inform and ultimately accelerate the development and application of non-invasive imaging technologies that can be applied to diagnosis, treatment and treatment planning, documentation of treatment response and indeed prediction of which individuals are likely to respond to a particular therapy. Further, the use of advanced functional imaging in companion species with spontaneous tumor-TME characteristics that better recapitulate human tumor-TME interplay would play an important role in novel tumor target identification, basic cancer growth and progression characterization, and the role of the immune system in cancer biology.

Much remains to be done to more successfully align and integrate companion species into cancer investigation pathways that involve advanced imaging. The harmonization of imaging protocols with consistent application of quality assurance and medical physics expertise is necessary to assure high quality and reproducible quantitative imaging in the comparative oncology setting. The integration of assurance endeavors such as those ongoing through the QIBA are essential to confidently interpret and apply the imaging data gathered in companion species. Additionally, some research tools readily available in rodent and human systems are currently lacking in companion species. In particular, immunologic and TME reagents that allow validation of immunomodulation and tumor-TME interactions are somewhat sparse in companion species; however, better organized and well-funded cooperative efforts such as the Immuno-Oncology Translational Network are rapidly expanding the available toolbox. Of course, no one model is perfect and not all research questions can be answered within the context of one model. In addition to the incomplete reagent toolbox, while some tumor histology's are genetically and phenotypically very similar between canines and humans (e.g., osteosarcoma) others have specific differences such as the near absence of BRAF mutations in canine melanoma. Such limitations, discussed in more detail in other manuscripts in this special volume of Frontiers, need to be considered when choosing a specific model to recapitulate human cancer biology. It is also not lost on the authors that the majority of examples compiled involve companion dogs and only one example of cats is presented. Unfortunately, companion cats have been relatively “orphaned” in the comparative oncology field in general and the advanced imaging area in particular. This may reflect several perceived limitations of the species, including smaller body size, clinical temperament, hepatic metabolism differences, species-specific reagent availability, and a lack of well-characterized histologies with human cancer correlates. The authors are aware of comparative trials about to begin that include cats with head and neck squamous cell carcinoma, a common cancer in the species and it is hoped that more attention will be paid the species in the comparative realm in the future.

The discussion and examples presented in this review serve to raise awareness of the utility of comparative oncology and companion species as a surrogate system that is ideal for bridging early preclinical small rodent investigations with clinical trials in humans.

## Author Contributions

DV, AL, and RJ conceived, produced, and edited this review article.

### Conflict of Interest

The authors declare that the research was conducted in the absence of any commercial or financial relationships that could be construed as a potential conflict of interest.
